# Suppressed Transmission of Long-Range Surface Plasmon Polariton by TE-Induced Edge Plasmon

**DOI:** 10.3390/mi12101198

**Published:** 2021-09-30

**Authors:** Guhwan Kim, Myunghyun Lee

**Affiliations:** Department of Electrical and Computer Engineering, Sungkyunkwan University, Suwon-si 16419, Korea; guhwankim@skku.edu

**Keywords:** plasmonics, surface plasmon polariton, edge plasmon, waveguide devices, plasmonic signal copier, nanoplasmonic integrated circuit

## Abstract

Work on controlling the propagation of surface plasmon polaritons (SPPs) through the use of external stimuli has attracted much attention due to the potential use of SPPs in nanoplasmonic integrated circuits. We report that the excitation of edge plasmon by TE-polarized light passing across gapped-SPP waveguides (G-SPPWs) leads to the suppressed transmission of long-range SPPs (LRSPPs) propagating along G-SPPWs. The induced current density by highly confined edge plasmon is numerically investigated to characterize the extended radiation length of decoupled LRSPPs by the TE-induced edge plasmon. The suppressed transmission of LRSPPs is confirmed using the measured extinction ratio of the plasmonic signals which are generated from the modulated optical signals, when compared to the extended radiation length calculated for a wide range of the input power. It is also shown that LRSPP transmission is sensitive to the excited power of edge plasmon in the gap through the permittivity change near the gap. Such a control of SPPs through the use of light could be boosted by the hybridized edge plasmon mode and a huge field enhancement using nanogap, gratings or metasurfaces, and could provide opportunities for ultrafast nano-plasmonic signal generation that is compatible with pervasive optical communication systems.

## 1. Introduction

Plasmonic materials, including metals below the plasma frequency, have an inherent ability to squeeze light within deep-subwavelength volumes through the kinetic energy of free carriers inside the material [1]. By virtue of this free electron contribution, the diffraction limit of light can be overcome [2]. Plasmonic materials have also been widely exploited in nanophotonic applications such as biosensing [3], lithography [4], display [5], second harmonic generation [6] and optical trapping [7]. Surface plasmons (SPs) are coherent charge density oscillations localized to the interface between the positive and negative permittivity materials, and they channel absorbed electromagnetic energy to free electrons. Further, resonant coupling between longitudinal SP waves and electromagnetic waves forms propagating surface plasmon polaritons (SPPs) along the interface [8].

SPPs are regarded as promising information carriers for the next generation of integrated circuits because a broad range of mode sizes can be realized through waveguide configurations [9,10,11] or phase control [12,13], and it can be detected on-chip directly, by electrical means [14,15] and by out-coupled (far-field) optical detection. The quantum properties of a photon-pair, including entanglement, can be preserved even in the conversion of photons into SPPs (photon-SPP), and vice versa (SPP-photon) [16,17]. A high bit-rate and the WDM data transmission over plasmonic waveguides were well demonstrated with low bit error rates [18,19] and there have been many efforts to develop waveguide-based plasmonic devices, for example the demultiplexer [20], a mode converter [12,21,22,23], a coupler [24,25], and a logic gate [26], as building blocks for nanoplasmonic integrated circuits (NPICs).

The control of the propagation of SPPs with external stimuli has attracted much attention for realizing SPP-based optical isolation, nonreciprocity and signal generation in NPICs. It has been found that drifting electrons using an external electric current [27,28,29,30,31] or static magnetic field [32,33,34] causes an asymmetric dispersion and the nonreciprocity of SPPs. In addition, the modulation of SPPs at the single metal–dielectric interface was demonstrated using a gate-biased field effect [35] and a light pulse [36,37]. In our recent study [38], we proposed that using a long-range SPP (LRSPP) as a carrier wave, a plasmonic signal can be invertedly copied from a modulated optical signal, through use of a plasmonic signal copier (PSC). Herein, we investigated the plasmonic response in the dielectric gap of a PSC for polarization power of the input optical signal, numerically and experimentally. Highly confined edge plasmon is only excited for TE-polarized optical signals, and induces strong electric currents inside the plasmonic waveguides, leading to the suppressed transmission of LRSPPs. Considering the dependence of the input optical power and the electric permittivity change on the dielectric material near the gap on plasmonic signals in a PSC, a discussion regarding the efficient control of SPPs with light is also presented.

## 2. Plasmonic Signal Copier and Long-Range Surface Plasmon Polariton

**Figure 1:**
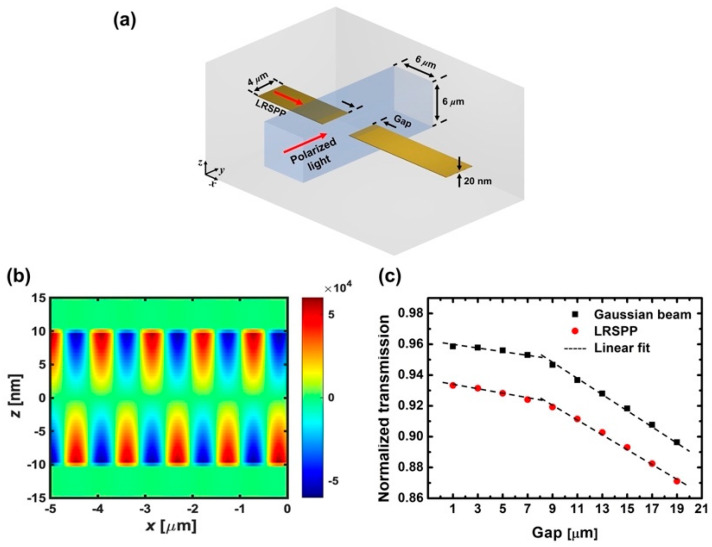
(**a**) 3D schematic of the plasmonic signal copier (PSC). (**b**) Induced current density (J*_x_*) by long-range surface plasmon polaritons (LRSPPs) in the propagation direction (unit: A/m^2^). (**c**) Comparison of the LRSPP transmission at the output surface plasmon polariton waveguide (SPPW) as a function of the gap size according to the type of excitation.

Figure 1a illustrates the 3D schematic of a PSC. A PSC is composed of gapped-surface plasmon polariton waveguides (G-SPPWs) [39,40] and a dielectric channel waveguide laid across the gap. The thickness of the gold SPP waveguides was 20 nm and low-loss polymers were used as the dielectric materials for the clad (n_clad_ = 1.45) and for the 6 × 6 μm^2^ core (n_core_ = 1.46) of the channel waveguide. Experiments were performed using 1.550 μm laser diodes. One of them was used to excite LRSPPs with a 20 nm thick input surface plasmon polariton waveguide (SPPW) with the power of 0 dBm, and the other was used to excite polarized optical signals with a variable optical attenuator and a polarization controller. Details on the experimental setup and fabrication processes can be found in [38,41]. To summarize, when LRSPPs and polarized optical signals intersected coincidently, a plasmonic signal, of which the binary state is inverted to the modulated optical signal, was detected for only the TE-polarized optical signal at the output SPPW rather than the TM-polarized optical signal. Using LRSPPs as carrier waves, a plasmonic signal is copied invertedly from the TE-polarized optical signal-like NOT gate (inverter).

The LRSPP excited in the input SPPW propagates along the *x* direction and carries an antisymmetric charge density distribution along the central horizontal plane of the waveguide. The excited LRSPP is one of the fundamental modes that is supported by a gold strip and has Gaussian-like field distribution. Figure 1b shows the surface plasmon wave of the LRSPP, characterized by the current density in the direction of propagation (J*_x_*). The current density is calculated using finite-difference time-domain (FDTD) simulations. The amounts of other components (J*_y_* and J*_z_*) are minute, due to the fact that J*_y_* contributes to the radiation in higher-order modes [42]; the normal component of the electric field (E*_z_*), proportional to J*_z_*, is significantly small inside the SPPW, due to the electromagnetic boundary condition [8,43]. At the end-facet of input SPPW, the guided LRSPP decouples to a TM-polarized lightwave (SPP-photon) due to the structural interruption that cannot allow the surface plasmon wave to be carried, and the Gaussian-like lightwave remains propagating along the direction of G-SPPWs. After the radiation in the gap, the lightwave re-excites the LRSPP (photon-SPP) at the front-facet of output SPPW through end-fire coupling [39,40,44,45], which implies that LRSPPs tunnel the dielectric gap via the SPP-photon-SPP conversion process. The transmission of LRSPPs decreases with the gap length, since the coupling loss at the front-facet of the output SPPW increases with the broadening of the Gaussian beam during the propagation in the gap, and the scattering also occurs at the edges of the channel waveguide.

To investigate why the transmission is lowered by the broadening of the Gaussian beam, the tunneling of LRSPPs across the gap and the coupling of LRSPPs by a Gaussian beam are compared. Figure 1c shows the FDTD-calculated transmission of LRSPPs at the output SPPW in the absence of an input optical signal across the gap. The red dots represent the transmission of the LRSPP when excited in the input SPPW (SPP-photon-SPP), and the black squares represent the transmission of the LRSPP when excited in the output SPPW after the radiation of the Gaussian beam in the gap. Without the input SPPW, the Gaussian beam is launched from the position of the end-facet of the input SPPW and thereby excites the LRSPP at the front-facet of the output SPPW (photon-SPP). The black dashed lines represent the linear trends of the transmission. The transmission is lowered linearly as the gap length increases. However, The slope which is the ratio of the transmission’s change to the gap length’s increment becomes steeper over the gap length of 8 μm for both cases as in G-SPPWs without the channel waveguide [39,40], due to the mode size mismatch between the diverging radiation mode and LRSPPs at the front-facet of output SPPW, increasing the photon-SPP coupling loss.

## 3. TE-Induced Edge Plasmon

**Figure 2:**
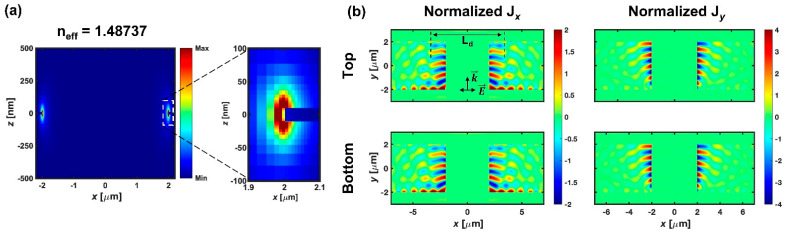
The finite-difference time-domain (FDTD) analysis of (**a**) the plasmonic mode excited by TE-polarized light in the gap (−2 μm ≤ *y* ≤ 2 μm) and (**b**) induced current densities (J*_x_* and J*_y_*) at the top and bottom interfaces of gapped-SPP waveguides (G-SPPWs) after normalization to the current density of the LRSPP.

We investigated the plasmonic response of G-SPPWs using FDTD analysis in the presence of incident polarized light across the 4 μm gap, as considered in the previous study [38]. When TE-polarized light is excited in the channel waveguide, its electric field oscillates parallel to the planar plane of the PSC (*x*-*y* plane) and accumulates electric charges at the end(front)-facet of input(output) SPPWs. As a result, the light excites a plasmonic mode in the gap. Figure 2a shows the FDTD-calculated cross-sectional mode profile of the excited mode in the gap due to the TE-polarized light. The inset shows the zoomed mode profile in the right edge (output SPPW). The edges of G-SPPWs are surrounded by the core of the channel waveguide (n_core_ = 1.46). Most of the fields are highly localized at the edges and the effective index is 1.48737, which indicates that the dispersion curve of the excited mode lies to the right of the respective light line, and an edge-guided plasmonic mode is excited in the gap by the TE-polarized light. Figure 2b shows the induced current density by the edge plasmon at the top and bottom interfaces of G-SPPWs. It is observed that the excited edge plasmon is a short-range SPP (SRSPP) [46], as the surface charge oscillates symmetrically at the top and bottom interfaces. The SRSPP exhibits apparently distinct characteristics from LRSPPs in field confinement and attenuation, inducing a strong current density. The induced current densities (J*_x_* and J*_y_*) are normalized to the amplitude of J*_x_* by LRSPPs (J_LRSPP_), to characterize the impact on the LRSPP propagation by the TE-induced edge plasmon. Far stronger current densities than that of the LRSPP are induced near the edges, and the symmetry of current density carried by the LRSPP would break if the LRSPP and TE-polarized light were incident to the gap coincidently. Therefore, the LRSPP propagating along the input SPPW radiates by scattering before it reaches the structural interruption (end-facet of the input SPPW). We defined the decoupling length (L_d_) as the summation of the gap length and the lengths for which the TE-induced current density is larger than that which is carried by the LRSPPs at the G-SPPWs. L_d_ represents the extended radiation length by TE-induced edge plasmon. The decoupled LRSPP by the edge plasmon radiates freely for the decoupling length and re-excites LRSPPs at the output SPPW, which leads to the suppressed transmission of LRSPPs by the extended radiation length.

**Figure 3:**
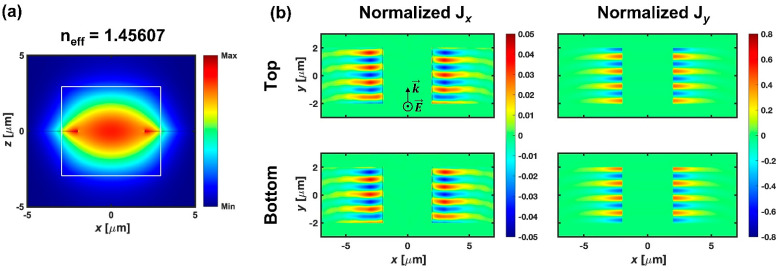
The FDTD analysis of (**a**) the photonic mode excited by TM-polarized light in the gap and (**b**) induced current densities (J*_x_* and J*_y_*) at the top and bottom interfaces of G-SPPWs after normalization to the current density of the LRSPP. Note that the scale of color bar is different with Figure 2b.

On the other hand, TM-polarized light exhibits quite a different response in the gap. The magnetic field of TM-polarized light excited in the channel waveguide oscillates parallel to the planar plane of PSC, and the electric field mainly oscillates in the *z*-axis. Figure 3a shows the cross-sectional mode profile of the excited mode in the gap by TM-polarized light, and the white solid line represents the 6 × 6 μm^2^ core of the channel waveguide. Most of the field is distributed in the core with a refractive index of 1.46. The mode profile is almost the same; the fundamental TM mode is excited in the channel waveguide and the effective index is 1.45607, which indicates that a photonic TM mode guided by the core is excited in the gap. Figure 3b shows the induced current density by the photonic mode at the top and bottom interfaces of G-SPPWs. Compared to the TE-induced current density, minute amounts of current density are induced, and the normalized value is also under 1, which implies that TM-induced current density cannot disturb the propagation of LRSPPs; therefore, all the power contained in incidental TM-polarized light passes across the gap in the photonic state without any disturbance to the LRSPP.

**Figure 4:**
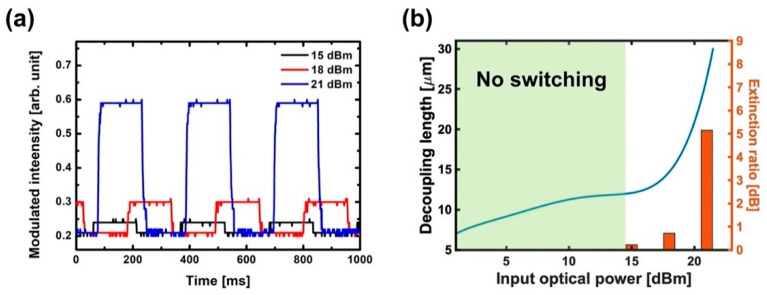
(**a**) Generated plasmonic signals by TE-polarized optical signals with the power of 15, 18 and 21 dBm in PSC. (**b**) Comparison between the calculated decoupling length and measured extinction ratio as a function of input optical power.

The suppressed transmission of LRSPPs by TE-induced edge plasmon was investigated experimentally for a wide range of the input optical power in PSC (0~21 dBm). The TE-polarized optical signals were modulated at 3 Hz frequency by the attenuator. Figure 4a shows the invertedly copied plasmonic signals from the TE-polarized optical signals with the power of 15, 18 and 21 dBm and the modulated intensities are normalized to the same minimum value. The extinction ratio, and on/off ratio of the copied plasmonic signal, increases with the input optical power. We also compared the calculated decoupling length to the measured extinction ratio of plasmonic signals (Figure 4b). The blue line represents the calculated decoupling length as a function of input optical power. The vertical bars represent the measured extinction ratio for the input optical power of 15, 18 and 21 dBm. As the input optical power increases, more power is transferred to free electrons to excite the edge plasmon, and a stronger current density is induced in the G-SPPWs, thereby extending the decoupling length. Copied plasmonic signals are observed in input powers of over 15 dBm, and the increasing extinction ratio also follows the dependence of power on the decoupling length. This indicates that the transmission difference of LRSPPs, between the ‘on’ and ‘off’ states of the input optical signal, increases with the power given that the ‘on’ state of the input optical signal leads to the suppressed transmission of LRSPPs through the excitation of edge plasmon. Such a comparison illustrates that the increasing extinction ratio of copied SPP signals is linked to the TE-induced current density parallel to LRSPP propagation. 

**Figure 5:**
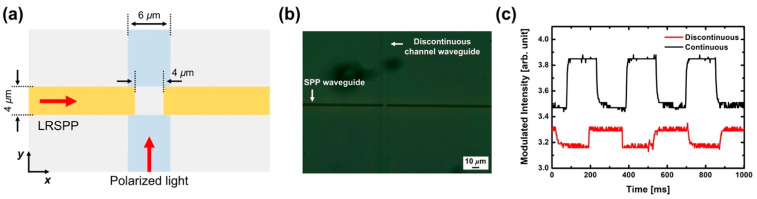
(**a**) Top view of the PSC with discontinuous channel waveguide in *x*-*y* plane. (**b**) Optical microscope image of the fabricated PSC sample with discontinuous channel waveguide. (**c**) Measured plasmonic signals for the continuous and discontinuous channel waveguide; the input optical power is 21 dBm.

We also investigated the dielectric effect on the PSC through the discontinuity of the channel waveguide. Figure 5a illustrates the top view of the PSC with a discontinuous channel waveguide and Figure 5b shows the optical microscope image of the fabricated PSC sample with a discontinuous channel waveguide. For such a structure, partial reflection and scattering occur at the end(front)-facet of the input(output) channel waveguide, and the effective index of excited edge plasmon in the gap is changed depending on the surrounding dielectric material. Figure 5c shows the copied plasmonic signals from TE-polarized optical signal with the power of 21 dBm for the continuous and discontinuous channel waveguides. It is found that the extinction ratio for the continuous channel waveguide is much larger than that of discontinuous one. The FDTD-calculated transmission of input light at the output channel waveguide does not exhibit distinct differences between the cases, which means that the reflection and scattering at the facets of the discontinuous channel waveguide do not affect the transmission of LRSPPs across the gap, due to the small index change. However, there is a significant difference observed in the excited edge plasmon mode. The effective indices of edge plasmon modes excited by the TE-polarized light in the gap for the continuous (discontinuous) channel waveguides are 1.48737 (1.48344). Due to the permittivity change of surrounding dielectric material and the power ratio of excited edge plasmon to the input optical power, P_edge_/P_input_ is 1.0084% for the continuous channel waveguides and 0.7867% for the discontinuous channel waveguides. Since the input optical power is the same for both cases, there are significant observable changes in the excited edge plasmon mode. Therefore, it is found that the transmission of LRSPPs across the gap is affected by the TE-induced edge plasmon, depending on the excited power of edge plasmon in the gap. This also explains the reason why a significantly larger input optical power is required to suppress the transmission of LRSPPs in a PSC. Therefore, it would be possible to improve the plasmonic signal generation efficiency by introducing plasmonic nanostructures so that more power is transferred to free electrons in order to excite the edge plasmon and induce a strong electric current parallel to the SPP propagation, reducing the overall power consumption. 

## 4. Discussion

We performed numerical FDTD analysis and experiments on the plasmonic response in the gap of a PSC with the polarization and power of an input optical signal, and for the discontinuous channel waveguide. The TE-polarized light excites the highly confined edge plasmon in the gap and induces a strong electric current inside the G-SPPWs, overwhelming the surface plasmon wave that is carried by the LRSPP, thereby breaking the symmetry at the top and bottom interfaces of G-SPPWs. The LRSPP decouples to a lightwave before it reaches the end-facet of the input SPPW and the transmission of the LRSPP is lowered by the extended radiation length and increased coupling loss. The decoupling length is defined in order to characterize the extended radiation length of the decoupled LRSPP by TE-induced edge plasmon, compared with the measured extinction ratio of plasmonic signals. For the PSC experiments with a wide range of input optical power, the extinction ratio of plasmonic signals increases with the input optical power, since more power is transferred to free electrons to excite the edge plasmon and a stronger current density is induced to the G-SPPWs, extending the decoupling length. Therefore, induced current density that is parallel to the SPP propagation can affect the propagation of SPPs [27,28,29,30,31]. We also considered a PSC with a discontinuous channel waveguide to investigate the dielectric effect. Although there is no significant difference in the transmission of input optical signal by the partial reflection and scattering, the characteristics and excited power of the edge plasmon exhibit distinct differences. In contrast to TE-polarized light, TM-polarized light does not excite the plasmon mode in the gap, and the excited photonic TM mode in the gap does not induce enough current density to disturb the LRSPP propagation by just passing through the gap, which is in agreement with the previous results that showed a plasmonic signal is not copied from a TM-polarized optical signal even with the power of 21 dBm [38]. Our findings suggest that the control of SPPs using light can be boosted by the enhancement of the induced electric current inside the plasmonic waveguide, which could be achieved by a large field enhancement in the nanogap, the plasmon hybridization [47] of edge plasmons at the left and right edges and by considering oblique incidence on gratings or metasurfaces in the gap. Furthermore, if it is integrated with a mode converter, it is possible to realize nano-plasmonic signal generation that is compatible with pervasive optical communication systems and a plasmonic inverter (NOT gate) in NPIC.

## 5. Conclusions

We show that the highly confined edge plasmon excited by TE-polarized light passing across G-SPPWs induces strong electric currents, resulting in the suppressed transmission of LRSPPs propagating along G-SPPWs. Considering the plasmonic response for the polarization, the power of the input optical signal and the discontinuous channel waveguide in a PSC, it is found that the extinction ratio of plasmonic signals increases as more power is transferred to free electrons used to excite highly confined edge plasmon. Such a control of SPPs with light could be boosted by the huge field enhancement and hybridized edge plasmon modes offered by nanogaps, gratings or metasurfaces and could provide opportunities for an ultrafast nano-plasmonic signal generation that is compatible with pervasive optical communication systems.
